# Chronic morphine and HIV-1 Tat promote differential central nervous system trafficking of CD3+ and Ly6C+ immune cells in a murine *Streptococcus pneumoniae* infection model

**DOI:** 10.1186/s12974-015-0341-5

**Published:** 2015-06-20

**Authors:** Raini Dutta, Sabita Roy

**Affiliations:** Department of Surgery, University of Minnesota, Minneapolis, MN 55455 USA; Department of Pharmacology, University of Minnesota, Minneapolis, MN 55455 USA

**Keywords:** Morphine, HIV-1 Tat, Systemic bacterial infection, Neuropathogenesis, Chemokine, TLR

## Abstract

**Background:**

Persistent systemic infection results in excessive trafficking of peripheral immune cells into the central nervous system (CNS), thereby contributing to sustained neuroinflammation that leads to neurocognitive deficits. In this study, we explored the role of opportunistic systemic infection with *Streptococcus pneumoniae* in the recruitment of peripheral leukocytes into the CNS and its contribution to HIV-1-associated neurocognitive disorders in opioid-dependent individuals.

**Methods:**

Wild-type B6CBAF1 (wt), μ-opioid receptor knockout (MORKO), FVB/N luciferase transgenic, and Toll-like receptor 2 and 4 knockout (TLR2KO and TLR4KO) mice were subcutaneously implanted with morphine/placebo pellet followed by HIV-1 Transactivator of transcription (Tat) protein injection intravenously and *S. pneumoniae* administration intraperitoneally. On postoperative day 5, brains perfused with phosphate-buffered saline were harvested and subjected to immunohistochemistry (for bacterial trafficking and chemokine ligand generation), flow cytometry (for phenotypic characterization of CNS trafficked immune cells), Western blot, and real-time PCR (for ligand expression).

**Results:**

Our results show differential leukocyte trafficking of T lymphocytes (CD3+) and inflammatory monocytes (Ly6C+) into the CNS of mice treated with morphine, HIV-1 Tat, and/or *S. pneumoniae*. In addition, we demonstrate a Trojan horse mechanism for bacterial dissemination across the blood-brain barrier into the CNS by monocytes. Activation of TLRs on microglia induced a chemokine gradient that facilitated receptor-dependent trafficking of peripheral immune cells into the CNS. HIV-1 Tat induced trafficking of Ly6C+ and CD3+ cells into the CNS; infection with *S. pneumoniae* facilitated infiltration of only T lymphocytes into the CNS. We also observed differential chemokine secretion in the CNS, with CCL5 being the predominant chemokine following HIV-1 Tat treatment, which was potentiated further with morphine. *S. pneumoniae* alone led to preferential induction of CXCL12. Furthermore, we attributed a regulatory role for TLRs in the chemokine-mediated trafficking of leukocytes into the CNS. Chronic morphine and HIV-1 Tat, in the context of systemic *S. pneumoniae* co-infection, differentially modulated induction of TLR2/4, which consequently facilitated trafficking of TLR2 → CD3 + CCR5+ and TLR4 → Ly6C+(CCR5+/CXCR4+) immune cells into the CNS.

**Conclusion:**

Our murine study suggests that secondary infection in opioid-dependent individuals infected with HIV-1 augments peripheral leukocyte trafficking as a consequence of sustained chemokine gradients in the CNS.

## Introduction

The prevalence of HIV-associated neurocognitive disorders (HAND) is on the rise because of longer survival rates resulting from antiretroviral therapy. These effects are more predominant in HIV-1-infected opioid-dependent individuals [1–4]. Furthermore, due to their immunosuppressive state, the incidence of systemic co-infection with opportunistic pathogens is also significantly higher in these patients. Frequent and repeated systemic infection in various neurodegenerative diseases, such as Alzheimer’s and Parkinson’s disease, results in unregulated activation of proinflammatory cytokines in the central nervous system (CNS) leading to progressive decline in cognitive function [5, 6]. Despite a significant body of literature documenting the association of recurrent systemic infection with increased neurocognitive deficits, its role in the prevalence of HAND in opioid-dependent individuals infected with HIV-1 has not been delineated.

Peripheral leukocyte migration into the CNS contributes to pathogenesis of inflammatory neurologic responses during systemic infection [7, 8] through induction of chemokines and their receptors. In the current study, we explored the contribution of specific chemokine receptors, i.e., CXCR4 and CCR5, in differential leukocyte trafficking into the CNS of mice following chronic morphine, HIV-1 Transactivator of transcription (Tat) protein, and/or co-infection with *Streptococcus pneumoniae*. CCR5 and CXCR4 are the predominant co-receptors that interact with CD4+ T cells for HIV-1 entry into host cells [9]. Additionally, various independent studies have shown that μ-opioid receptor (MOR) agonists, including morphine, exacerbate the expression of CXCR4 and CCR5 on peripheral immune cells, as well as on microglial cells, thereby increasing HIV-1 infectivity [10–14].

Toll-like receptors (TLRs) on various immune cells recognize conserved motifs expressed by pathogens and play an important role in recruiting leukocytes to the sites of infection [15–17]. Unregulated TLR activation has been implicated in HIV-1 pathogenesis [18, 19]. We also previously showed that TLR activation on microglial cells results in significant neuronal apoptosis in a HIV-1 Tat model with co-infection of *S. pneumoniae* in opioid-dependent mice [20]. In the present study, we expanded our previous observations to further investigate the role of TLR-mediated CNS leukocyte trafficking as a contributing mechanism for HAND following chronic exposure to morphine in a co-infection murine model.

We demonstrated, in a murine model, morphine treatment in the context of HIV-1 Tat and *S. pneumoniae* orchestrate the migration of T cells through induction of chemokine ligands CXCL12 and CCL5. Furthermore, we attribute a specific role for TLR2 and TLR4 in the chemokine-mediated leukocyte trafficking into the CNS. In summary, secondary bacterial co-infection in a murine model of HIV-1 infection and drug abuse exacerbated peripheral immune cell trafficking, thus disrupting neuroimmune homeostasis, thereby contributing to HAND.

## Materials and methods

### Experimental animal

Experiments were conducted on 8- to 12-week-old male mice. Wild-type B6CBAF1 (wt), μ-opioid receptor knockout (MORKO), FVB/N luciferase transgenic, and Toll-like receptor 2 and 4 knockout (TLR2KO and TLR4KO) mice were obtained from Jackson Laboratory (Bar Harbor, ME) and maintained in pathogen-free animal housing facilities with a constant temperature (22 ± 1 °C) and humidity (50 %) and with a regulated 12-h light/dark cycle. Animals were housed three mice per cage and given standard food and tap water ad libitum. Animal studies were approved by the Institutional Animal Care and Use Committee at the University of Minnesota. All procedures are in agreement with the guidelines set forth by the National Institutes of Health’s *Guide for the Care and Use of Laboratory Animals.*

### Treatment

Mice were treated according to the procedure explained in our previously published paper with slight modification [20]. Briefly, mice anesthetized with isoflurane (Halocarbon Products Corp., River Edge, NJ) were subcutaneously implanted either with placebo or with a slow-release morphine (25 mg, NIDA, Rockville, MD) pellet (blood levels are 0.9–1.6 μg/ml at steady state) and then injected intravenously (i.v.) with recombinant full-length HIV-1 Tat protein (82 aa, Immunodiagnostic Systems Inc., Gaithersburg, MD) with a dose of 10 μg/kg [20–22]. To model an opportunistic infection in these mice, animals were inoculated intraperitoneally (i.p.) with *S. pneumoniae* serotype 3 strain (American Type Culture Collection, ATTC 6303, Manassas, VA) with a dose of 1 × 10^3^ colony-forming units (CFUs) in phosphate-buffered saline (PBS; 0.01 M, 500 μl) 24 h following morphine treatment. Five days following infection, animals were sacrificed and tissues were harvested for ex vivo analysis.

### Bioluminescence imaging and bacterial translocation

Morphine- or placebo-treated mice were inoculated intraperitoneally with luciferase-tagged *S. pneumoniae* serotype 3 (Xen10, Xenogen Corporation, Alameda, CA) at a dose of 1 × 10^3^ CFUs/500 μl PBS in the presence or absence of HIV-1 Tat protein. At day 5, in live animals, bacterial clearance and dissemination into the CNS were imaged using Xenogen’s IVIS CCD camera system [23]. Total photon emission from selected and defined areas within the images of each mouse was quantified as photons/s/cm^2^ using Living Image (Xenogen) and Igor (WaveMetrics, Lake Oswego, OR) image analysis software.

### Luciferase activity

To measure luciferase activity in brain tissues, we used a luminometer (TD-20/20, Turner Designs Inc., Sunnyvale, CA). Animals were placed under gas anesthesia using isoflurane (2–2.5 %) and cardially perfused with ice-cold PBS (0.01 M). After perfusion, animals were sacrificed and PBS-perfused brains were aseptically removed and homogenized in lysis buffer (Promega Corporation, Madison, WI). After centrifugation at 10,000 *g* for 10 min, the supernatant of the brain lysate was collected and mixed with the substrate luciferin (1:4; Promega Corporation); luminescence was measured for 15 s by the luminometer. The total protein concentration of the brain homogenates was determined by a bicinchoninic acid (BCA) protein assay kit (Thermo Fisher Scientific Inc., Rockford, IL) to normalize luciferase activity.

### Adoptive transfer

Translocation of immune cells into the CNS of animals treated with or without morphine, HIV-1 Tat protein, and *S. pneumoniae* was examined using adoptive transfer experiment. HIV-1 Tat protein (10 μg/kg) [20, 22] was given i.v*.* in age- and sex-matched donors, FVB/N luciferase transgenic mice (luciferase expression driven by the β-actin promoter; Xenogen Corporation, Alameda, CA) and recipient mice (B6CBAF1), after subcutaneous morphine (25 mg) and placebo pellet implantation. Twenty-four hours post pellet implantation, *S. pneumoniae* (1 × 10^3^ CFUs/500 μl) was given i.p. The spleens were aseptically removed from FVB/N luciferase transgenic mice at day 4 and splenocytes were adoptively transferred into wild-type mice as described elsewhere [24]. Briefly, the harvested spleens were homogenized and red blood cells were lysed using ammonium chloride lysing reagent. Mixed luciferase-positive immunocytes (1 × 10^7^ cells per mouse) were transferred via tail vein injection into the recipient mice at day 4. After 24 h, luciferase substrate (D-luciferin, 150 μg, Gold Biotechnology, St. Louis, MO) was administered by i.p. injection, mice were imaged using Xenogen’s IVIS CCD camera system, and data were acquired using a 5-min exposure window. The total photon emission of each mouse was quantified as described in the “Bioluminescence imaging and bacterial translocation” section. Further, luciferase activity in brain tissues was measured as described in the “Luciferase activity” section.

### Immunohistochemistry

Prefrontal cortex (PFC) brain regions perfused with ice-cold PBS were divided into three different tissue sections and snap frozen in liquid nitrogen for different experiments, confocal imaging, RT-PCR, and Western blot. In HIV/AIDS infection, the PFC is especially important; it undergoes disease-associated changes in synaptic tone that produce abnormal neurocognitive phenotypes which strongly resemble many of the behavioral anomalies that occur in HAND [25–27]. For immunohistochemistry experiments, brain coronal cryostat (Leica Microsystems Inc., IL) sections (5 μm) were prepared from groups of snap-frozen brain tissue. For intracellular immunostaining, cryostat tissue sections were fixed with 4 % paraformaldehyde, then permeabilized with 0.2 % PBS-Triton X, and blocked with PBS-BSA (5 %) [25–27] for 1 h at room temperature. Slides were washed with PBS-Tween (PBS-T; 0.02 %) and subsequently stained with required antibodies.

To identify the cellular or acellular mode of *S. pneumoniae* trafficking into the CNS, confocal microscopy was used. Mouse anti-CD45, anti-CD3, and anti-Ly6C (BD Biosciences; dilution 1:500) and rabbit anti-*S. pneumoniae* (Serotec; dilution 1:500) were used as primary antibodies. Alexa Fluor 488-conjugated anti-mouse IgG (Invitrogen; dilution 1:1000) and Rhodamine Red goat anti-rabbit IgG (Jackson ImmunoResearch Laboratories; dilution 1:1000) were used as secondary antibodies.

Chemokine ligands (CXCL12 and CCL5) and their co-localization with glia, astrocytes, and microglia were also observed using confocal microscopy. Mouse anti-CCL5 and mouse anti-CXCL12 were used as primary antibodies (eBioscience; dilution 1:200), and Alexa Fluor 488-conjugated anti-mouse IgG (Invitrogen; dilution 1:1000) was used as a secondary antibody. To identify glia, Alexa Fluor 555-conjugated glial fibrillary acidic protein antibody (GFAP; Cell Signaling Technology; dilution 1:1000) was used as an astrocyte marker and rabbit anti-ionized calcium-binding adapter molecule 1 (IBA-1; Wako, CA; dilution 1:200) was used as a microglia marker. Rhodamine Red goat anti-rabbit IgG was used as a secondary antibody for microglia detection (Jackson ImmunoResearch Laboratories).

After staining with specific antibodies, tissues were washed with PBS-T and mounted using ProLong Gold antifade reagent with 4′,6′-diamidino-2-phenylindole (DAPI; nuclei marker; Invitrogen; dilution 1:5000) and dried overnight in the dark. Immunofluorescence images were obtained on a Nikon Eclipse Ti confocal microscope using a ×60 oil immersion objective. Sectioning was performed on a minimum of five random sections from each of six individual mice per experiment.

### Isolation of brain leukocytes and flow cytometry

For immunophenotyping of brain-infiltrated leukocytes, we used flow cytometry. At the end of the treatment period, mice were lethally anesthetized using CO_2_ asphyxiation and the brains were removed after being cardially perfused with ice-cold PBS as described in the “Luciferase activity” section. Brain tissue was cut into small pieces and enzymatically digested using collagenase (Liberase, Roche) and single-cell suspension separated by conventional 40/70 Percoll centrifugation. Mononuclear single cells were incubated with purified rat anti-mouse CD16/CD32 (2.4G2) (BD Biosciences) for 10 min at 4 °C to reduce the nonspecific binding to the Fcγ receptor. Cells were subsequently incubated for 30 min at 4 °C with monoclonal antibodies (10 μg/ml) ACPCy7-CD45 (30-F11), PECy7-CD11b (M1/70), PerCP-CD3 (145-2C11), and phycoerythrin-conjugated Ly6C (AL-21) (all from BD Biosciences).

To detect chemokine receptors on CNS immune cells, we used biotinylated CCR5 (C34-3448) and CXCR4 (2B11/CXCR4) antibodies (10 μg/ml) as primary antibodies; the secondary antibody was streptavidin conjugated with PerCP/PE. To stain TLRs on immune cells, we used Alexa Fluor 488-conjugated anti-mouse TLR4 (UT41) and FITC-TLR2 (6C2) (eBioscience; dilution 1:200). The cells were washed twice and resuspended in 200 μl PBS with 0.1 % sodium azide. Dead cells were excluded by TROPO3 (Invitrogen) staining, and specific isotype controls (BD Biosciences) were used in parallel. Cells (10,000 events) were acquired using a FACSCanto II cytometer, and data were analyzed using Diva software (BD Biosciences).

### RT-PCR

Total RNA was extracted from frozen perfused brain tissue using TRIzol reagent (Life Technologies, Carlsbad, CA). RNA was reverse-transcribed using random hexamers from the TaqMan Reverse Transcription Reagents and RT Reaction Mix (Applied Biosystems, Inc., Foster City, CA) in a total volume of 40 μl at 25 °C for 10 min, at 42 °C for 30 min, and at 94 °C for 5 min on a PCR machine (Mastercycler gradient, Eppendorf, Germany). The resulting cDNA was used as a template for RT-PCR. Specific oligonucleotide primer sequences used in the amplification of chemokine ligand genes were CCL5 and CXCL12 (Integrated DNA Technologies, Inc, Coralville, IA). Details of primer sequences are given in Table 1. Real-time PCR was performed using SYBR Green Master Mix (Applied Biosystems) on the ABI Prism 7500 sequence detection system (Applied Biosystems). 18S rRNA served as an internal control in relative RT*-*PCR*.* The relative levels of chemokine ligands in the brain homogenate were quantified using the ΔCt-ΔCt method and were expressed as the percentage-fold change.

**Table 1:** Primers used for quantitative real-time RT-PCR

Primer	Forward (5'–3')	Reverse (5'–3')
CCL5	CCC TCA CCA TCA TCC TCA CT	TCC TTC GAG TGA CAA ACA CG
CXCL12	TCA GTG GCT GAC CTC CTC TT	TTT CAG CCA GCA GTT TCC TT
18S	TTG ACG GAA GGG CAC CAC CAG	CTC CTT AAT GTC ACG CAC GAT TTC

### Western blot

Semi-quantitative estimation of CCL5 and CXCL12 chemokine ligand levels in the mouse brain was done using Western blot. Proteins were harvested from frozen brain tissues using RIPA cell lysis buffer (Biotech Corporation). The protein concentrations were measured by using BCA protein assay kit. Equal aliquots of total SDS-soluble proteins (100 μg) were resolved to 15 % discontinuous SDS-PAGE. The transferred nitrocellulose membranes were blocked with 5 % fat-free milk in PBS-T (blocking buffer) at room temperature for 1 h and then incubated with primary antibodies (mouse anti-CCL5 and rabbit anti-CXCL12 or anti-β-actin antibody (eBioscience; 1:1000 dilution) at 4 °C overnight. After incubating with primary antibodies, the membranes were washed with PBS-T three times. Then the membranes were incubated for 1 h with IRDye 800CW-conjugated goat anti-rabbit IgG and IRDye 680-conjugated goat anti-mouse IgG secondary antibodies (LI-COR Biosciences, Lincoln, NE) diluted in blocking buffer. The blots were then washed three times with PBS-T and rinsed with PBS. Proteins were visualized by scanning the membrane on Odyssey Infrared Imaging System (LI-COR Biosciences) with both 700- and 800-nm channels. ImageJ software was used to quantify the band intensity. The protein levels of CCL5 and CXCL12 in the CNS were represented by the ratios of optical densities in their bands normalized against β-actin.

### Statistical analysis

SPSS 16.0 statistical software was used for statistical analysis. Data were collected from three independent experiments and expressed as mean ± SEM. Statistical significance was calculated by one-way ANOVA followed by Bonferroni’s multiple-comparison test among different groups. For correlation analysis, the Spearman rank order correlation coefficient was used. Results were considered significant at *P ≤* 0.05.

## Results

### Dissemination of *S. pneumoniae* into the CNS significantly increased in morphine-treated mice exposed to HIV-1 Tat protein

Several previous studies including ours have shown that morphine-induced immunosuppression results in lack of pathogen clearance in the infected animals [20, 23, 28, 29]. An increased pathogen burden results in their dissemination to various compartments, including the CNS.

**Fig. 1:**
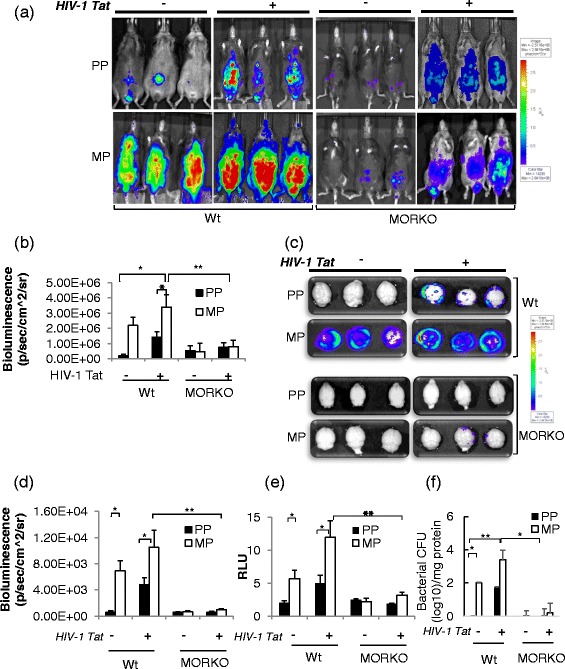
Significant increase in bacterial translocation into the CNS of mice chronically treated with morphine and HIV-1 Tat and co-infected with *S. pneumoniae* at the 5th day of post-pellet implantation. **a** Real-time in vivo bioluminescence imaging of luciferase-tagged *S. pneumoniae* (serotype 3) administered (i.p.) in live wild-type (*wt*) and μ-opioid receptor knockout (*MORKO*) mice chronically treated with morphine and HIV-1 Tat. **b** Histograms of mean bioluminescent signals from luciferase-tagged *S. pneumoniae* quantified as total photon emission from treated live mice. **c**, **d** Bacterial translocation into the CNS was detected in perfused mice brain using Xenogen’s IVIS CCD camera system, and total photon emission was quantified using Igor image analysis software. **e** Luciferase activity was measured in the whole brain tissue dissected out from treated mice. Brain lysates were isolated by homogenizing the brain tissue, and luciferase activity was measured using a luminometer, TD-20/20. Relative luminescence units (*RLU*) were normalized with the *Renilla* luciferase values. **f** Histograms of mean bacterial counts in brain tissue. *Error bars* indicate the standard error of the mean (SEM) of six different animals from each group (*n* = 6). *Pseudocolor scales* are shown to *right* of the figures. *PP* placebo pellet, *MP* morphine pellet. *P < 0.05; **P < 0.01

In our current study, using real-time in vivo bioluminescent imaging of live mice infected with luciferase-tagged *S. pneumoniae* (serotype 3), we showed a significant increase in bacterial load when they were treated with morphine alone. The effect was further exacerbated in mice treated with both morphine and HIV-1 Tat. Increased bacterial load indicative of reduced bacterial clearance resulted in severe bacteremia in the morphine-treated animals in the presence or absence of HIV-1 Tat (Fig. 1a, b). The present data is consistent with our previous findings, where we reported that morphine treatment results in lack of bacterial clearance when *S. pneumoniae* was administered intranasally [20]. Current data in combination with the previous observation suggest that bacterial inoculation given either i.p. or intranasally results in a significant increase in bacterial load in morphine-treated animals, despite the route of infection.

Next, we investigated if the lack of bacterial clearance increased bacterial trafficking into the CNS. PBS-perfused mouse brains were aseptically dissected, and the intensity of the luciferase signal was measured using Xenogen imaging. Placebo-treated mice efficiently cleared bacterial infection from the periphery; however, in the morphine-treated group, either in the presence or in the absence of HIV-1 Tat, a significantly higher luciferase intensity was observed in the brain, indicative of significantly greater bacterial translocation into the CNS (Fig. 1c, d). To further confirm that the photon emission by luciferase signaling was CNS derived, PBS-perfused brains from the respective groups were homogenized and the luciferase activity of luciferase-tagged *S. pneumoniae* was evaluated from the brain lysate using a luminometer. Morphine treatment showed significantly higher luciferase activity in the brain lysate which is further exacerbated with HIV-1 Tat (Fig. 1e).

Importantly, the synergistic increase in bacterial load (Fig. 1f) in the morphine-treated wt mice was significantly attenuated in the morphine-treated MORKO mice (Fig. 1c–e). These observations validate the significant role of μ-opioid receptors in morphine-mediated bacterial trafficking. Further, as expected, HIV-1 Tat-induced bacterial trafficking into the CNS in the wt animals was similar to that in the MORKO animals.

### Peripheral immune cell trafficking into the CNS significantly increased in mice chronically treated with morphine in the context of HIV-1 Tat and *S. pneumoniae*

**Fig. 2:**
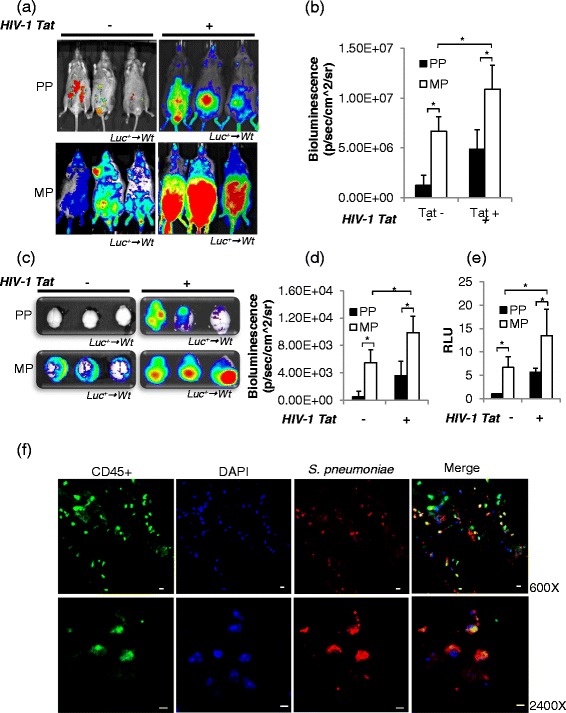
Massive increase of immune cell trafficking into the prefrontal cortex of the CNS of mice treated with morphine and HIV-1 Tat and systematically infected with *S. pneumoniae* at the 5th day of post-pellet implantation. **a** Splenocyte homing was assessed following the adoptive transfer of luciferase-expressing immune cells (1 × 10^7^) into B6CBAF1 recipient mice. The recipient mice were imaged for whole-body bioluminescence at 24 h following the transfer of luciferase-positive immune cells. **b** The mean bioluminescent signal was measured as total photon emission using Igor image analysis software as explained in Fig. 1b. **c** Immune cell homing in the perfused brain was assessed and **d** measured as explained in Fig. 1. **e** Luciferase activity in the brain lysate was measured (indicative of the number of immune cells) and is presented as histograms of six different animals from each group. Relative luminescence units (*RLU*) were normalized with the *Renilla* luciferase values. *Error bars* indicate the standard error of the mean (SEM) of six different animals from each group (*n* = 6). *Pseudocolor scales* are shown to *right* of the figures. **f** Confocal microscopy and z-stacked images showing CD45+ immune cells (Alexa Fluor 488-conjugated anti-mouse IgG; *green*) in the prefrontal cortex brain tissue with *S. pneumoniae* (Rhodamine Red goat anti-rabbit IgG; *red*) and counterstained nuclei with DAPI (*blue*), suggesting cellular mode for bacterial translocation into the CNS of mice treated with morphine and HIV-1 Tat and co-infected with *S. pneumoniae*. Merged images show co-localization of CD45+ immune cells and *S. pneumoniae*. Magnification ×600 (*upper panel*) and ×2400 (*lower panel*). *PP* placebo pellet, *MP* morphine pellet. *Scale bar* 10 μm. *P < 0.05

Next, we investigated if peripheral immune cells in the presence of morphine and/or HIV-1 Tat in the context of secondary bacterial co-infection results in increased trafficking. To test this hypothesis, an adoptive cell transfer experiment was performed as described in the “Materials and methods” section. The bioluminescence of adoptively transferred luciferase*-*positive immune cells was significantly higher both at the site of bacterial infection and in the CNS of morphine- and/or HIV-1 Tat-treated wt mice when compared to placebo-treated mice (Fig. 2a–e)*.* Our data strongly suggests that chronic morphine in the context of systemic Gram-positive bacterial infection and HIV-1 Tat exposure induces significant migration of peripheral immune cells into the CNS.

### Cellular mode of *S. pneumoniae* trafficking into the CNS of mice chronically treated with morphine and HIV-1 Tat protein and infected with *S. pneumoniae*

Bioluminescence imaging of live animals and their respective brains showed significantly higher bacterial load with subsequent higher immune cell trafficking into the CNS of mice treated with morphine and HIV-1 Tat and co-infected with *S. pneumoniae*. Our results support the proposed “Trojan horse” mechanism for the trafficking of infected immune cells across the blood-brain barrier (BBB) with subsequent release of pathogen within the CNS [30, 31]. To determine if *S. pneumoniae* trafficking into the CNS was occurring through infected peripheral immune cells as a cellular mechanism or through direct noncellular mechanism across endothelial cells, we investigated co-localization of *S. pneumoniae* with CD45+ antigen (leukocyte common antigen), a unique and ubiquitous membrane glycoprotein, expressed on almost all hematopoietic cells except erythrocytes and platelets. The presence of *S. pneumoniae* in the CNS of immune cells was observed through immunohistochemistry of brain tissue sections of animals treated with morphine, HIV-1 Tat protein, and *S. pneumoniae.* Co-localization of CD45+ immune cells with *S. pneumoniae* suggests a cellular mode of bacterial trafficking into the CNS of the treated mice (Fig. 2f), and this observation supports a Trojan horse mechanism for bacterial transmigration through BBB in our model of systemic *S. pneumoniae* infection.

### Immunophenotyping of live CD45+ immune cells revealed a significant increase in CD3+ and Ly6C+ immune cell population in the CNS of treated mice

**Fig. 3:**
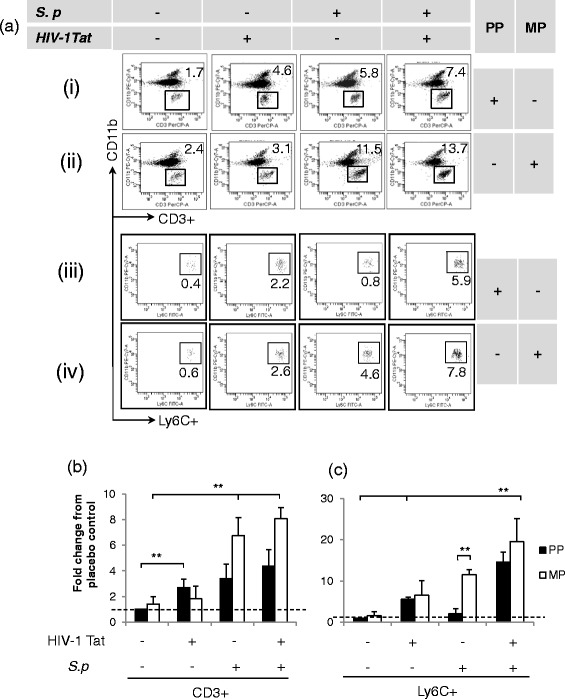
Significant trafficking of CD3+ and Ly6C+ immune cell population into the CNS of mice treated with morphine and HIV-1 Tat and co-infected with *S. pneumoniae* (*S.p*) at the 5th day of post-pellet implantation. Single-cell suspension was isolated from the perfused brain of treated mice. Flow cytometry analysis was performed to measure the frequency of TOPRO3-CD45_high_CD11b-CD3+ (T cells) and TOPRO3-CD45_high_CD11b+Ly6C+ (inflammatory monocytes) in the CNS. **a** Representative dot plots demonstrating high influx of T lymphocytes and inflammatory monocytes into the CNS of treated mice. The *histogram* represents quantification of specific immune cell (**b** CD3+; **c** Ly6C+**)** frequency as fold change from the group of untreated mice [basal level (*dashed line*)] in the CNS. *Error bars* indicate the standard error of the mean (±SEM) of six animals in each group (*n* = 6 mice). *PP* placebo pellet, *MP* morphine pellet. **P < 0.01

We observed differential leukocyte trafficking in the CNS of mice following treatment with morphine, HIV-1 Tat, and *S. pneumoniae*. Multicolor *phenotypic* characterization of TOPRO3-CD45_high_-gated cells (live immune cells) revealed ~3-fold increase in CD11b-CD3+ (T lymphocytes) and ~6-fold increase in CD11b+Ly6C+ (inflammatory monocytes) into the CNS of mice treated with HIV-1 Tat alone (Fig. 3a (i), (iii) and Fig. 3b, c). Systemic bacterial infection alone resulted in a significant increase of only CD3+ lymphocytes (~3-fold) into the CNS of treated mice. Chronic morphine does not induce any immune cell trafficking by itself; however, in the presence of *S. pneumoniae*, it significantly upregulated the trafficking of T lymphocyte (~5-fold) and inflammatory monocyte (~8-fold) population into the CNS of treated mice (Fig. 3a (ii), (iv) and Fig. 3b, c). Importantly, when all three treatment groups were present, a robust infiltration of both populations was observed (CD3 ~ 8-fold; Ly6C ~20-fold) (Fig. 3)*.* This result suggests that chronic opioid and HIV-1 Tat following systemic co-infection with *S. pneumoniae* result in a dramatic trafficking of both inflammatory monocytes and T lymphocytes into the CNS which may then contribute to unregulated inflammatory cytokine release which can potentially damage healthy bystander brain cells and disrupt CNS homeostasis as reported earlier [32–34].

### CXCR4 receptor expression on T lymphocytes and inflammatory monocytes promoted their trafficking into the CNS in morphine-treated mice exposed to HIV-1 Tat and *S. pneumoniae*

To identify the specific mechanism for immune cell trafficking into the CNS, we investigated the expression level of chemokine receptors on immune cells (CD3+ and Ly6C+) that migrated into the CNS. We chose two important chemokine receptors, CCR5 and CXCR4, as these are the co-receptors used for HIV-1 viral entry.

**Fig. 4:**
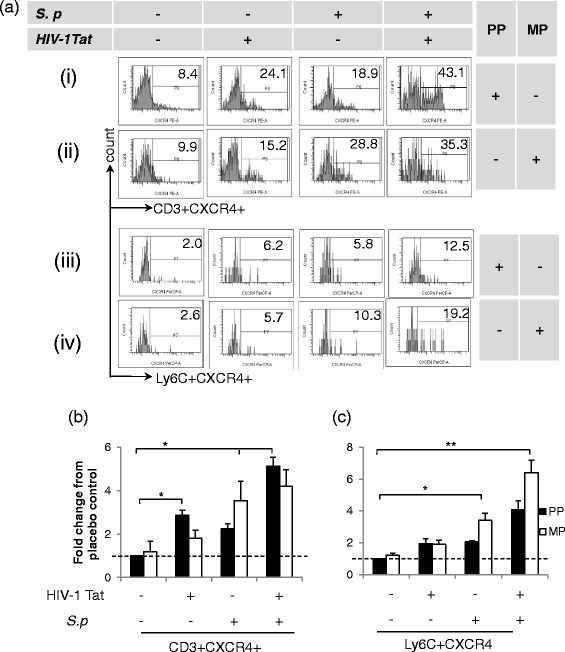
Enhanced chemokine expression of CXCR4 on immune cells isolated from the perfused brain of mice treated with morphine, HIV-1 Tat, and *S. pneumoniae* (*S.p.*) at the 5th day of post-pellet implantation. Flow cytometry profile of single-cell suspension. **a** Representative histograms demonstrate CXCR4 expression on leukocytes isolated from the CNS of treated mice. Histograms demonstrating CXCR4 expression (*i* and *ii*) on CD3+ and (*iii* and *iv*) on Ly6C+ cells. Bar diagrams representing quantification of CXCR4 expression on **b** CD3+ and **c** Ly6C+ cells as a mean fold change from placebo control [basal level (*dashed line*)]. *Error bars* indicate the standard error of the mean (SEM) of six different animals from each group (*n* = 6). *PP* placebo pellet, *MP* morphine pellet. *P < 0.05; **P < 0.01

**Fig. 5:**
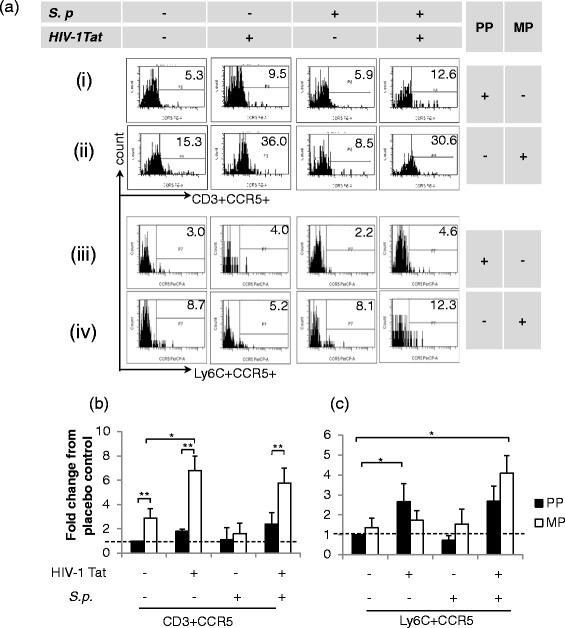
Enhanced chemokine expression of CCR5 on immune cells isolated from the perfused brain of mice treated with morphine, HIV-1 Tat, and *S. pneumoniae* (*S.p*) at the 5th day of post-pellet implantation. Flow cytometry profile of single-cell suspension. **a** Representative histograms demonstrating CCR5 expression on a distinct immune cell. Histograms demonstrating CXCR4 expression (*i* and *ii*) on CD3+ and (*iii* and *iv*) on Ly6C+ cells. Bar diagrams representing quantification of CCR5 expression on **b** CD3+ and **c** Ly6C+ as a mean fold change from the group of mice implanted with placebo pellets [basal level (*dashed line*)]. *Error bars* indicate the standard error of the mean (SEM) of six different animals from each group (*n* = 6). *PP* placebo pellet, *MP* morphine pellet. *P < 0.05; **P < 0.01

Systemic *S. pneumoniae* infection in the presence of both morphine and HIV-1 Tat led to significant CXCR4 expression on CD3+ T lymphocytes which is consistent with their migration pattern into the CNS (Fig. 3a, b), suggesting that CXCR4 expression is involved in T cell migration (Fig. 4a (i), (ii) and Fig. 4b)*.* Interestingly, although we did not observe significant trafficking of CD3+ T lymphocytes into the CNS of morphine-treated mice with HIV-1 Tat, CCR5 expression did significantly increase, suggesting that the chemokine gradient required for CCR5-induced trafficking might not be activated under these conditions (Fig. 5a (ii) and Fig. 5b).

Morphine treatment alone did not induce either trafficking of the inflammatory monocytes or the expression of CCR5 and CXCR4 (Figs. 4 and 5). However, significant trafficking of the monocytic population was observed in the presence of systemic infection with *S. pneumoniae* and*/*or with HIV-1 Tat treatment with a concurrent increase in CXCR4 expression. These data suggest that trafficking of both CD3+ T lymphocytes and inflammatory monocytes mediated through CXCR4 and not through CCR5 receptors*.* These data further suggest that systemic infection might potentiate inflammatory monocyte trafficking into the CNS of drug-dependent individuals (Figs. 4 and 5)*.*

We show in Fig. 3 that HIV-1 Tat treatment alone results in significant trafficking of inflammatory monocytes (~6-fold). In Fig. 4, we show a significant increase in CCR5 expression following HIV-1 Tat treatment, suggesting a dominant role for CCR5 in HIV-1-induced inflammatory monocyte trafficking. Importantly, treatment of animals with all three insults, i.e., morphine, HIV-1 Tat, and *S. pneumoniae* significantly upregulates the expression of both CCR5 and CXCR4 on inflammatory monocytes and on CD3+, indicating the participation of both receptors in the trafficking of peripheral immune cells (Figs. 4 and 5).

### *S. pneumoniae* used Ly6C+ cells as Trojan horse for trafficking into the CNS

**Fig. 6:**
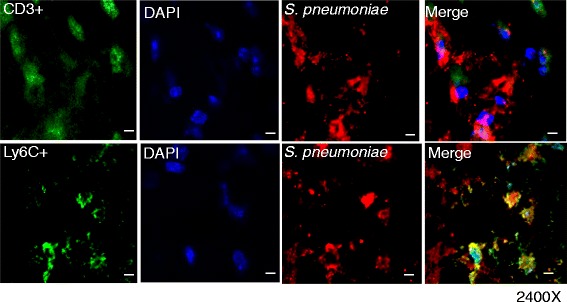
Ly6C+ cells transport *S. pneumoniae* across the blood-brain barrier. Confocal microscope and z-stacked images of mouse prefrontal region brain cryostat sections (5 μm) showing co-localization of immune cells, CD3+ and Ly6C+ cells (Alexa Fluor 488-conjugated anti-mouse IgG; *green*), with *S. pneumoniae*. Merged images show co-localization of *S. pneumoniae* (Rhodamine Red goat anti-rabbit IgG; *red*) with Ly6C+ cells. Nuclei were counterstained with DAPI (*blue*). Magnification ×2400. *Scale bar* 10 μm

To characterize the specific immune cells which carry pathogen across BBB into the CNS, perfused brain cryostat tissue sections from mice chronically treated with morphine, HIV-1 Tat protein, and *S. pneumoniae* were immunostained with specific cellular markers*.* We observed co-localization of *S. pneumoniae* with inflammatory monocytes (Ly6C+), suggesting the presence of intracellular bacteria in the monocyte population (Fig. 6). Positive staining of CD3 antigen confirmed the presence of T lymphocytes in the mouse brain. However, we did not observe co-localization of *S. pneumoniae* with CD3+ T lymphocytes (Fig. 6). The results suggest that dissemination of *S. pneumoniae* into the CNS is through a cellular mechanism and mediated through a monocyte subset. Additionally, our flow cytometry data also showed high monocyte trafficking into the CNS of mice chronically treated with morphine following *S. pneumoniae*, validating our finding of increased cellular bacterial trafficking into the CNS (Fig. 3).

### Systemic *S. pneumoniae* infection regulated TLR expression in immune cell subtypes following morphine treatment and in the context of HIV-1 Tat and *S. pneumoniae*

Activation of monocytes via TLR2 or TLR4 agonists could differentially modulate immune cell chemokine receptor expression and, therefore, potentially modulate recruitment of specific immune cells [17, 35]. Our previous published data documented an increase in microglial TLR (TLR2 and TLR4) surface expression in mice treated with morphine, HIV-1 Tat, and *S. pneumoniae* [20]. Additionally, we reported significant attenuation of the bacterial load into the CNS of TLRKO mice treated similarly to wt mice, suggesting a crucial role of TLRs in CNS leukocyte trafficking. Therefore, in the present study, we investigated the role of TLRs in the recruitment of immune cell into the CNS of mice treated with chronic morphine and HIV-1 Tat following systemic infection with *S. pneumoniae*.

**Fig. 7:**
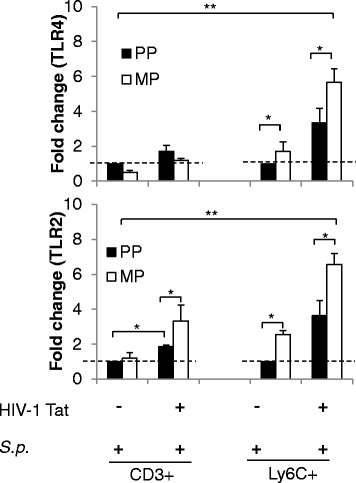
Differential surface expression of Toll-like receptors (TLRs) on distinct immune cells at the 5th day of post-pellet implantation. Histograms demonstrate mean fold change in TLR4 and TLR2 expression on the CD3+ and Ly6C+ immune cells in the CNS prefrontal cortex region of mice treated with morphine, HIV-1 Tat, and *S. pneumoniae. Error bars* indicate the standard error of the mean (SEM) of six different animals from each group (*n* = 6 mice). *PP* placebo pellet, *MP* morphine pellet. *P < 0.05; **P < 0.01

In the presence of HIV-1 Tat and systemic *S. pneumoniae* co*-*infection, chronic morphine treatment resulted in a significant increase in both TLR4 and TLR2 expressions on inflammatory monocyte (Ly6C+) cell population (Fig. 7). However, on CD3+ cells, only TLR2 expression was significantly upregulated (Fig. 7).

### Trafficking of peripheral immune cells into the CNS was TLR dependent

We next investigated if differential upregulation of TLRs is responsible for the differential immune cell trafficking pattern. A differential increase in TLR expression on immune cells that trafficked into the CNS was observed in groups of mice chronically treated with morphine and HIV-1 Tat protein following infection with *S. pneumoniae* (Fig. 7). We evaluated the role of selective TLR activation in the trafficking of peripheral immune cells into the CNS of the treated animals.

**Fig. 8:**
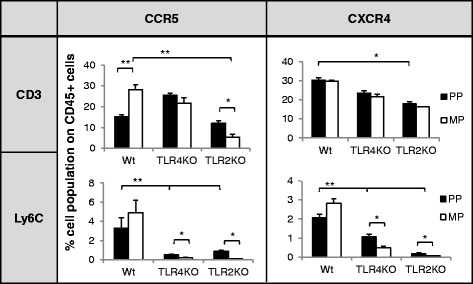
Differential trafficking of peripheral leukocytes with chemokine receptors (*CCR5* and *CXCR4*) at day 5 into the CNS of wild-type (*wt*) and TLR knockout (*TLRKO*) mice treated with morphine and HIV-1 Tat and systemically infected with *S. pneumoniae*. Histogram of percentage mean leukocyte (*CD3*+ and *Ly6C*+) trafficking with CCR5 and CXCR4 into the CNS of wt and TLRKO mice. *Error bars* indicate the standard error of the mean (SEM) of six different animals from each group (*n* = 6). *PP* placebo pellet, *MP* morphine pellet. *P < 0.05; **P < 0.01

In the presence of HIV-1 Tat, morphine-induced CCR5 expression and *S. pneumoniae*-induced CXCR4 expression on CD3+ T lymphocytes were downregulated only in the TLR2KO mice. However, CXCR4 expression on inflammatory monocytes (Ly6C+) was dramatically downregulated in both TLR2KO and TLR4KO mice with or without chronic morphine treatment. These results implicate the significant role of TLR2 in CD3+ cell migration and both TLR2 and TLR 4 in the trafficking of inflammatory monocytes (Fig. 8).

### Chemokine ligands CCL5 and CXCL12 were significantly upregulated in the CNS of mice chronically treated with morphine and HIV-1 Tat and infected with *S. pneumoniae*

To determine if the cognate ligands for the upregulated chemokine receptors (CCR5 and CXCR4) are also modulated in the brain tissues of mice treated with morphine, HIV-1 Tat, and *S. pneumoniae*, protein as well as the mRNA levels of respective chemokine ligands CCL5/RANTES (Regulated upon Activation, Normal T-cell) and CXCL12/SDF1 (stromal cell-derived factor-1) were evaluated in brain homogenates [16].

**Fig. 9:**
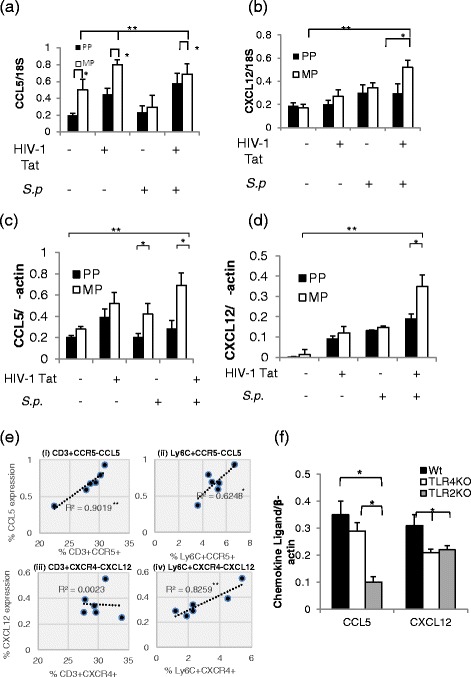
Chemokine ligand (CCL5 and CXCL12) production is significantly high in the prefrontal cortex region of mice chronically treated with morphine, HIV-1 Tat, and *S. pneumoniae*. Histograms of differential mRNA expression of **a** CCL5 and **b** CXCL12 genes using 18S mRNA as internal control in prefrontal cortex brain tissue isolated from the treated mice from all the groups. Histograms of band intensity of chemokine protein synthesis of **c** CCL5 and **d** CXCL12 using ImageJ software. **e** Correlation analysis (*R* ^2^) of CNS ligand (CCL5 and CXCL12) expression on the *y*-axis with peripheral immune cell trafficking (*i* and *iii*) CD3+ and (*ii* and *iv*) Ly6C+ into the CNS (*x*-axis) of the wild-type animals treated with chronic morphine, HIV-1 Tat, and *S. pneumoniae*. **f** Histograms of mean quantification of chemokine ligand CCL5 and CXCL12 generation in the prefrontal cortex region of wild-type and TLR knockout mice. *Error bars* indicate the standard error of the mean (SEM) of six different animals from each group (*n* = 6). *PP* placebo pellet, *MP* morphine pellet. *P < 0.05; **P < 0.01

Interestingly, HIV-1 Tat protein in the presence or absence of chronic morphine treatment significantly induced CCL5 generation; however, synthesis of CXCL12 was specifically upregulated by bacterial treatment (Fig. 9). Importantly, in the presence of all three insults, i.e., morphine, *S. pneumoniae*, and HIV-1 Tat protein, a dramatic increase in both ligands was observed. Importantly, CNS ligand expression positively correlates with immune cell (CD3+ and Ly6C+) trafficking into the CNS of treated mice (Fig. 9e). In morphine-treated mice following HIV-1 Tat protein and *S. pneumoniae* infection, CCL5 expression in the CNS resulted in significant trafficking of CD3+ and Ly6C+ population (Fig. 9e (i), (ii)); however, CXCL12 expression correlates with trafficking of only Ly6C+ cells (Fig. 9e (iv)). Data suggest that chemokine gradient facilitates a receptor-dependent trafficking of peripheral immune cells into the CNS.

Furthermore, to confirm whether the decrease in peripheral immune cells trafficking into the CNS of TLRKO is associated with decrease in brain chemokine production, immunoblotting of total protein isolated from the brain tissue of wt, TLR2KO, and TLR4KO mice was performed. We observed a significant decrease in CXCL12 synthesis in both TLR2 and TLR4KO mice; however, the level of CCL5 was significantly lower only in the TLR2KO mice (Fig. 9f). Our data implicates that functional TLRs are essential for chemokine ligand synthesis. We previously documented that morphine in the presence of *S. pneumoniae* and HIV-1 Tat protein increased TLR expression on human cells as well as on murine microglial cells [20]. Here, we established that upregulation of TLR expression modulates chemokine production, thereby generating a gradient for the trafficking of peripheral immune cells with specific chemokine receptors into the CNS.

### Morphine treatment in the context of HIV-1 Tat and *S. pneumoniae* infection increased chemokine ligand (CCL5 and CXCL12) synthesis by microglial cells

**Fig. 10:**
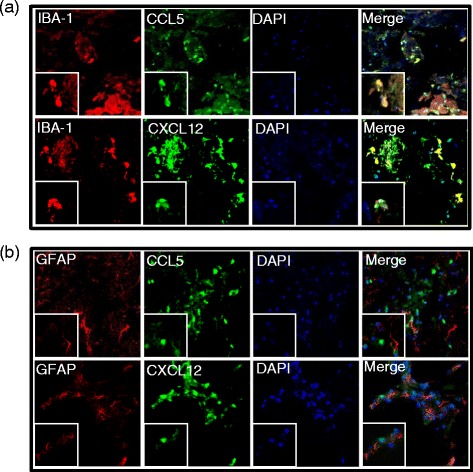
Co-localization of chemokine ligands and microglia. Confocal microscope and z-stacked images of the prefrontal cortex brain tissue region showing co-localization of chemokine ligands (Alexa Fluor 488-conjugated anti-mouse IgG; *green*): **a** CCL5 and **b** CXCL12 (Rhodamine Red goat anti-rabbit IgG; *red*) with microglia (IBA+) rather than astrocytes (GFAP+). Counterstained nuclei of the cells with DAPI (*blue*). Magnification ×600. Merged images present co-localization of glia (microglia or astrocytes) with chemokine ligand (CCL5 or CXCL12). *Scale bar* = 10 μm. Magnification of the *boxed area* ×2400

To identify the cell type that produced the chemokine ligands, microglia cells were stained for IBA1 and astrocytes for GFAP and their co-localization with CCL5 and CXCL12 evaluated. Results show that both the chemokine ligands, i.e., CCL5 and CXCL12, co-localized with IBA1 but not with GFAP, revealing microglia as the major source of chemokine production in the brain tissue of mouse treated with morphine, HIV-1 Tat protein, and *S. pneumoniae* (Fig. 10).

## Discussion

Opioid use and abuse accelerates HIV-1-associated neurocognitive disorders (HAND) [36, 37]. Peripheral leukocyte recruitment into the CNS has been implicated in neuropathogenesis associated with neuroAIDS [3, 8, 20, 32, 33, 38, 39]. However, the detailed mechanism underlying this process is unclear. The exacerbated induction of proinflammatory mediators from activated leukocytes into the CNS through repetitive episodes of systemic infection contributes to neurocognitive deficits often observed in neurodegenerative diseases, such as Alzheimer’s and Parkinson’s [5, 40, 41].

In the current study, we demonstrate that chronic administration of morphine either independently or together with HIV-1 Tat protein resulted in persistent bacterial infection in mice. This finding is consistent with our earlier findings where opiate abuse resulted in decreased bacterial clearance and increased susceptibility to opportunistic infection [42]. In addition, in our current study, we also delineated the specific roles of morphine and HIV-1 Tat on differential TLR expressions that contribute to distinct leukocyte recruitment into the CNS following systemic bacterial infection. We also demonstrate a Trojan horse mechanism for bacterial dissemination across the blood-brain barrier into the CNS by monocytes.

Morphine treatment results in attenuated bacterial killing following *S. pneumoniae* infection. The regulatory mechanism associated with this outcome is either impaired TLR9-NF-κB signaling or blunted phagocytic properties of immune cells following exposure to morphine [23, 43]. Compromised bacterial killing increased bacterial load and resulted in persistent systemic infection. Morphine induced inhibition of systemic bacterial clearance leading to increased bacterial burden following their dissemination into the CNS which was significantly abolished in the MORKO mice, suggesting that morphine-mediated augmentation of *S. pneumoniae* infection was mediating through μ-opioid receptors. Furthermore, in wild-type animals, we observed higher bacterial dissemination into the CNS of mice treated with HIV-1 Tat alone. A possible mechanism for the increased bacterial dissemination with HIV-1 Tat-treated animals is significant disruption of blood-brain barrier integrity following HIV-1 Tat treatment, as reported earlier [44].

Furthermore, recent studies by Olin et al. [45] show profound effects on splenocyte infiltration into the CNS in an inflammation model in the presence of chronic morphine. Their finding is consistent with our present finding where we observed a significant infiltration of peripheral immune cells into the CNS of animals treated with morphine and/or HIV-1 Tat protein following systemic infection with *S. pneumoniae*. The characterization of phenotypic markers on live immune cells revealed a differential influx of T lymphocytes (CD3+) and inflammatory monocytes (Ly6C+) into the CNS*.* HIV-1 Tat alone resulted in CNS trafficking of both CD3+ T lymphocytes and Ly6C+ inflammatory monocytes. Surprisingly, systemic bacterial infection resulted in dramatic trafficking only of T lymphocytes. Although morphine treatment alone did not alter the trafficking of any immune cell, we observed robust and synergistic CNS trafficking of both T cells and inflammatory monocytes when morphine-treated animals were exposed to HIV-1 Tat and co-infected with *S. pneumoniae*.

T lymphocytes are considered to be neuroprotective in CNS damage, but their deleterious role in myelin degradation in various autoimmune diseases has also been reported [46, 47] through activation of inflammatory cytokines such as IFN-γ and IL-17. We previously reported that the synergistic effect of chronic morphine with *S. pneumoniae* and HIV-1 Tat increased proinflammatory cytokine (TNF-α, IL-6, and MCP-1) synthesis in the CNS [20]. We now demonstrate exacerbated T cell and inflammatory monocyte recruitment into the CNS following morphine, *S. pneumoniae*, and HIV-1 Tat treatment, suggesting that migrated immune cells contribute to the inflammatory milieu, disrupting CNS hemostasis, eventually resulting in neuropathology.

The contributing role of chemokines and their receptors in potentiating leukocyte trafficking during infection is well documented. In the present study, we demonstrated that HIV-1 Tat alone resulted in increased CCR5 expression on T lymphocytes with their subsequent trafficking into the CNS. We further showed that systemic bacterial infection modulated the trafficking of both T lymphocytes and monocytes by activating the expression of both CXCR4 and CCR5. However, the activation of chemokine receptors alone is not sufficient for the migration of leukocytes. Rather, a highly regulated network of chemokine gradient was needed for directing cell trafficking. Exaggerated production in the microglia of the cognate ligands (CXCL12 and CCL5) for the corresponding chemokine receptors (CXCR4 and CCR5) provided chemical gradients for attracting peripheral leukocytes to the site of expression. Detailed analyses of our data revealed that morphine induced an increase in CXCL12 generation in the presence of systemic infection. However, the generation of CCL5 is predominantly a HIV-1 Tat effect. When all three insults are on board, a synergistically increased production of both chemokine ligands in the CNS resulted in a massive infiltration of activated peripheral immune cells into the brain. Excessive leukocyte recruitment with surplus chemokine production in the CNS has been shown to trigger neuronal injury and death via overactivation of p38 MAPK [48].

Surprisingly, we found that chronic morphine, either alone or with HIV-1 Tat, did not result in the trafficking of any immune cells, even though it significantly increased the surface expression of CCR5 on T lymphocytes. A potential explanation lies in the heterodimerization and cross-desensitization of the chemokine CCR5 receptor with μ-opioid receptors in the presence of morphine, which can alter the efficiency of ligand binding and signaling, thereby contributing to decreased chemotaxis [49]. Moreover, it has been previously shown that CD3+ T lymphocyte trafficking is mediated primarily through the CXCR4 chemokine receptor [9]. In our study, morphine, primarily, in the presence of HIV-1 Tat downregulated both CXCR4 expression and the production of its cognate ligand, CXCL12, in the CNS, thereby explaining the decreased T cell trafficking into the CNS, although the expression of CCR5 and its cognate ligand was high.

Numerous studies including ours have suggested that morphine modulates various intracellular signaling elements of TLR pathways or directly binds to MD-2, thereby inducing TLR4 oligomerization and activating TLR4 signaling [20, 50, 51]. We previously demonstrated that morphine potentiated TLR2 and TLR4 expression on microglial cells leading to significant induction of proinflammatory cytokines with a concurrent increase in reactive oxygen species, nitric oxide production, and caspase-3 activation, thereby contributing to neuronal damage [20]. In the current study, we demonstrated that chronic morphine in the presence of HIV-1 Tat and *S. pneumoniae* resulted in TLR2 activation on T lymphocytes (Fig. 7). However, we observed induction of both TLR2 and TLR4 on inflammatory monocytes. Activated TLRs induce upregulation of proinflammatory transcription factors (e.g., NF-κB, activator protein 1 (AP-1), signal transducer and activator of transcription (STAT)) which drives promoters of proinflammatory cytokines and chemokines that play a critical role in the pathophysiology of neuroAIDS [18, 19, 52].

To the best of our knowledge, the present study is the first to clearly demonstrate the distinct role of TLRs in chemokine-induced peripheral leukocyte trafficking into the CNS of HIV-infected opioid-dependent animals particularly in the presence of systemic pneumonia infection. Data shows that chronic morphine treatment in the presence of HIV-1 Tat resulted in significant CNS trafficking of Ly6C+CCR5+ and Ly6C+CXCR4+ following co-infection with systemic *S. pneumoniae* infection, an effect that was significantly attenuated in both TLR2KO and TLR4KO mice. Significantly less monocyte (the bacterial transporters) trafficking into the CNS results in reduced *S. pneumoniae* dissemination into the CNS of TLR2KO and TLR4KO mice as reported previously [20]. Further, the generation of cognate chemokine ligands CCL5 and CXCL12 is significantly attenuated in both TLR2KO and TLR4KO mice. HIV-1 Tat treatment activates TLR2 induction of CCL5 ligand and mediates the CNS trafficking of CD3+CCR5+ T lymphocyte. Till date, HIV-1 Tat protein is reported to activate TLR4 on monocytes which contribute to abnormal hyper-activation of the immune system via TNF-α production [53]. The present investigation suggests that TLR2 activation by HIV-1 Tat on T lymphocytes might also play a significant role. Further, TLR2 signaling upregulates T cell infiltration and microglial expansion through the MyD88-dependent pathway [35].

Taken together, our study shows that chronic morphine treatment results in significant upregulation of TLR2 and TLR4 expression, both on peripheral immune cells and on microglia. Subsequent activation of TLRs by HIV-1 Tat and/or *S. pneumoniae* in morphine-treated mice results in upregulation of chemokine receptor expression on peripheral immune cells, with a concurrent increase in their cognate ligand secretion by microglial cells. A combination of receptor and ligand expression in different compartments induces migration of peripherally activated peripheral leukocytes into the CNS. The resulting exacerbated neuroinflammatory responses might be a contributing factor in HIV-1-associated neuropathogenesis observed in the drug-abusing population that are HIV positive. These results also indicate that systemic infection with Gram-positive bacteria may potentiate neuropathogenesis in this population. Therapeutic interventions that target TLR activation could be targeted to mitigate neuroinflammation associated with neuroAIDS.
